# Fungal Infection and Inflammation in Cystic Fibrosis

**DOI:** 10.3390/pathogens10050618

**Published:** 2021-05-18

**Authors:** T. Spencer Poore, Gina Hong, Edith T. Zemanick

**Affiliations:** 1 Department of Pediatrics, University of Colorado Anschutz Medical Campus, Aurora, CO 80045, USA; spencer.poore@childrenscolorado.org; 2 Department of Medicine, Perelman School of Medicine, University of Pennsylvania, Philadelphia, PA 19104, USA; Gina.Hong@pennmedicine.upenn.edu

**Keywords:** fungus, *Aspergillus*, co-infection, cystic fibrosis, allergic bronchopulmonary aspergillosis, inflammation

## Abstract

Fungi are frequently recovered from lower airway samples from people with cystic fibrosis (CF), yet the role of fungi in the progression of lung disease is debated. Recent studies suggest worsening clinical outcomes associated with airway fungal detection, although most studies to date are retrospective or observational. The presence of fungi can elicit a T helper cell type 2 (Th-2) mediated inflammatory reaction known as allergic bronchopulmonary aspergillosis (ABPA), particularly in those with a genetic atopic predisposition. In this review, we discuss the epidemiology of fungal infections in people with CF, risk factors associated with development of fungal infections, and microbiologic approaches for isolation and identification of fungi. We review the spectrum of fungal disease presentations, clinical outcomes after isolation of fungi from airway samples, and the importance of considering airway co-infections. Finally, we discuss the association between fungi and airway inflammation highlighting gaps in knowledge and future research questions that may further elucidate the role of fungus in lung disease progression.

## 1. Introduction

Cystic Fibrosis (CF) is one of the most common inherited conditions, resulting in early morbidity and shortened life expectancy [1]. Cystic fibrosis is caused by absence or dysfunction of the cystic fibrosis transmembrane conductance regulator (CFTR) protein which primarily affects the pulmonary and gastrointestinal systems [1]. Lack of CFTR protein in the epithelial cell membrane impairs chloride and bicarbonate movement at the cell surface, resulting in thickened secretions in the airways, pancreas and other organs [2]. Manifestations start early in life and pulmonary complications are the most common causes of morbidity and mortality as people with CF (pwCF) age. Individuals with CF are prone to persistent airway infections due to dehydrated mucus that accumulates in the airways [3,4]. This abnormal mucus creates a protein rich environment for organisms to grow, leading to chronic infection and resulting in inflammatory response [5,6]. This in turn leads to airway obstruction, remodeling and bronchiectasis, causing a progressive decline in lung function.

Common culprits for airway infection in CF are bacteria found in the environment. *Pseudomonas aeruginosa*, *Staphylococcus aureus*, and other gram-negative bacteria are frequently detected from airway samples and chronic infection with these pathogens associated with worse outcomes (e.g., lower lung function, increased frequency of pulmonary exacerbation) [7,8,9,10,11,12]. Pulmonary exacerbations, episodic increases in respiratory symptoms characterized by decreased lung function, increased cough and increased sputum production contribute to much of the morbidity in pwCF [13,14].

In addition to bacteria, fungi are also frequently detected in airway samples; yet, their contribution to lung disease progression is not well understood [15]. Fungal infections may be transient or chronic, although here has yet to be consensus on how to define chronic infection from these organisms [15,16,17]. Furthermore, fungi are difficult to culture from CF airway samples, making detection and true prevalence difficult to determine [18,19]. Transient detection of fungus with subclinical airway changes, fungal bronchitis, sensitization to fungal proteins, and allergic bronchopulmonary aspergillosis (ABPA) have all been described, yet clear consensus guidelines have yet to be determined [15,17]. Studies have shown an association with worse clinical outcomes in some individuals with fungi, but whether fungi are independent contributors to poor outcomes or a reflection of a diseased airway is not clear [20,21,22].

Allergic bronchopulmonary aspergillosis is an allergic inflammatory process leading to airway obstruction and airway remodeling [23,24]. As opposed to CF endo-bronchitis associated with bacterial pathogens, ABPA is caused by an allergic response to fungal elements rather than true infection. In pwCF, ABPA increases pulmonary morbidity, yet how it develops and who is at risk are not well understood [23]. Here we review the spectrum of fungal disease presentations, clinical outcomes after isolation of fungi from airway samples, and the importance of considering airway co-infections in CF. We also discuss the association between fungi and airway inflammation highlighting gaps in knowledge and future research questions that may further elucidate the role of fungi in lung disease progression in CF.

## 2. Epidemiology

Airway fungal recovery is common in pwCF. *A. fumigatus* and other *Aspergillus* species are the most frequently detected fungi with reported prevalence ranging from 10–50%, although there is a variety in patient populations and clinical laboratory practices that impacts detection rates as reported by multiple reviews and studies [15,16,25,26]. In an analysis of 2012 registry data from Germany among 2600 pwCF, 32% isolated fungus and the prevalence of *Aspergillus* and *Candida* isolation also increased with age. Hong et al. used United States CF Foundation Patient Registry (CFFPR) data from 2006–2012 to evaluate the individual level factors associated with *Aspergillus* infection in pwCF [27]. They found that pancreatic insufficiency, chronic anti-*Pseudomonas* aerosolized antibiotics, inhaled corticosteroid use, and macrolide antibiotic use were risk factors for recurrent *Aspergillus* isolation [28]. Similar to a single center study, these data suggest that advancing disease and therapeutic exposures disrupting the bacterial microenvironment may increase the likelihood of isolating fungus from the airway [29]. There appears to be an inflection point of likely fungus isolation around adolescence and early adulthood, although fungus may be isolated in younger populations [30,31]. In an Australian pediatric cohort that underwent early bronchoscopy, *Aspergillus* was a frequently isolated organism in infants and young children and associated with radiographic changes correlating with early lung disease [30]. Such findings challenge the argument that *A. fumigatus* is present without pathogenicity. Lower rates of fungi reported in young populations may be related to an inability to detect fungus in those who cannot adequately expectorate sputum, or to disease stage.

*Scedosporium* has also been identified in airway samples from pwCF [19,22,32,33,34]. Recent German registry data noted that *Scedosporium* was one of the more common fungi isolated from pwCF (1.9% of pwCF in 2017 registry) [22]. *Scedosporium* has been found in approximately 3% of pwCF in the United States and, similar to *Aspergillus*, has been associated with older age, inhaled antibiotic use, and increased IV antibiotic episodes [32]. Yeast including *Candida albicans* are also detected with estimated prevalence of 50–75%, although laboratory reporting of yeast varies [19,22]. Other fungal pathogens that may have clinical consequences in CF include *Rasamsonia argillacea* complex, *Trichosporon mycotoxinovorans*, and *Exophiala dermatitidis*; however, the rarity of these organisms leads to difficulty interpreting observed clinical correlations [35,36,37,38].

## 3. Microbiologic Detection

In pwCF, bacterial cultures are routinely obtained from the airway at each clinic visit and at the time of pulmonary exacerbation. Whether cultures are obtained using oropharyngeal swabs (OP), sputum, or bronchoalveolar lavage depends on the patient and other factors, comorbidities, and clinical status. Sputum cultures are ideal, as mucus presumably represents many areas of the lung. If people cannot spontaneously expectorate sputum, OP swabs are obtained in attempts to survey what organisms may be present in the lower airways, or sputum induction is attempted [39,40,41]. Bronchoscopy with bronchoalveolar lavage (BAL) allows sampling of various lobes of the lung but the procedure is invasive and often requires anesthesia; thus, its use for surveillance is limited [42]. While surveillance of airway bacterial cultures is recommended at least quarterly and with illnesses as part of standard clinical care, the optimal frequency of culturing for fungi is not known [43].

Fungi can be difficult to detect from airway cultures. While OP swabs are routinely used for bacterial surveillance in non-expectorating individuals, fungal detection from upper airway samples is difficult to interpret and is not routinely recommended [43,44]. Furthermore, OP swab detection is not always representative of lower airway disease [45]. Yield of fungal detection from OP swabs is low and often does not accurately represent whether fungus is present or not in the lower airway [19,46]. Since this occurs, pwCF who do not produce sputum and whose pathogen screening has been limited to OP swabs, may have undetected fungus in the lower airways. Fungal cultures from sputum and bronchoalveolar lavage specimens should be performed using fungal-specific media or selective media enriched with antimicrobials to promote fungal growth. However, fungus can remain difficult to isolate due to differences between species in optimal media, incubation temperatures, processing, and time for growth [44,47]. Coron et al. found that by using specific and multiple culture plates, 81% of expectorating individuals with CF isolated fungus or yeast [19]. However, this tiered research study approach is likely not available in clinical laboratories. In studies using molecular approaches, 60% of the fungal genus and species detected were not found on routine fungal culture [48]. Furthermore, Delhaes et al. reported differences in fungal prevalence by culture conditions and media, age and geography, and recommended standardized approaches across laboratories [25].

Given the limitations of fungal detection with microbiologic culture approaches, investigators have applied microbial sequencing methods to study the mycobiome in CF airway specimens [49]. Molecular techniques use sequencing approaches targeted towards common fungal genomes, identifying fungi without the need for culture [50]. Using these approaches, species previously undetected are being found in the CF airway. Yet, the clinical effect of such findings is still under investigation, as well as the utility of molecular detection versus classic culturing methods [18,49]. Further work regarding the detection of fungus either by culture media, PCR testing, or next-generation sequencing is needed.

Regarding the detection of host responses to fungi, advanced animal and cell-based studies have investigated fungus and the inflammatory response. Toor et al. investigated transcriptomic inflammatory pathway responses of human bronchial epithelial cells to *A. fumigatus* conidia and found a variety of host gene responses (autophagy, complement, coagulation, cytokine receptor interactions, and chemokine responses, etc.) as well as 1% internalization of conidia by human bronchial epithelial cells [51]. Other studies have investigated conidia binding of *A. fumigatus*, internalization of spores and epithelial cell participation in host response, identifying inflammatory pathways and responses to these interactions within the airway [52,53,54]. Other investigators have expanded this to genomic studies as well as to studies evaluating nasal airway responses [55,56]. While there are few CF specific studies, these early studies provide a unique approach to understanding host response to inhaled fungi. These pathways may help develop other methods of detecting fungi in the airway and provide clinical metrics beyond current culture methods.

## 4. Pathophysiology

In pwCF, fungal infections are likely more to be common due to the unique properties of CF mucus and reduced mucociliary clearance [57]. Fungi are likely to be inhaled as conidia, or non-germinated spores. The conidia are small and inhaled diffusely into the lower airways, where goblet and mucus producing cells reside. These spores are inhaled daily by most people and do not cause infection as they are exposed to immune cells and thin mucous that is expelled via normal breathing and the mucociliary escalator [57]. In CF, however, the mucous is thick and dehydrated. Conidia become lodged within the sputum and are difficult to expectorate [16]. This environment allows the conidia to germinate, creating hyphae and complex cellular structures. As this occurs, the fungi may utilize nutrients, cell free DNA, and other biofilm contents from other organisms to thrive and grow within the mucous and airways [58,59]. Given the complex mucus network and viscosity, inflammatory response cells have a difficult time reaching these organisms in the CF airway. This in turn results in an unregulated growth response, allowing the fungi to thrive and attack the epithelial cells of the airway. By the time the inflammatory cells of the host are exposed to the fungus, it has grown hyphae and formed a mature structure, making it hard to kill and remove from the pulmonary system [60]. This likely results in inflammatory change of the airway mucosa, alveolar damage and, with some fungal species, vascular invasion in specific hosts [16,27].

CFTR modulators, including ivacaftor, lumacaftor/ivacaftor, tezacaftor/ivacaftor and elexacaftor/tezacaftor/ivacaftor, improve the function of CFTR on the cell surface, improving chloride and bicarbonate transport through various mechanisms depending on the underlying mutation [61]. Mucociliary clearance has been shown to improve following ivacaftor therapy for pwCF and a G551D-CFTR mutation [62]. Heltshe et al. found that, in patients treated with ivacaftor, *Aspergillus* detection and isolation was reduced following treatment [63]. Whether this is related to less sputum production, less infection, or other factors has yet to be determined. As elexacaftor/tezacaftor/ivacaftor has now been approved for most pwCF, longitudinal studies to determine the impact of treatment on fungal infections will provide important information for future management [64].

## 5. Spectrum of Fungal Infection in CF

In pwCF, recovery of fungi may reflect colonization or infection with resultant inflammatory response and detrimental impact on airways disease (sometimes termed “fungal bronchitis”). Fungi may also be detected in the setting of ABPA, an allergic Th2-mediated reaction to inhaled fungal proteins, as well as fungal sensitization without meeting ABPA criteria. The most frequent presentations associated with fungal recovery and disease in pwCF are discussed below.

### 5.1. Persistent Infection and Transient Infection

Some pwCF isolate fungus yet show little signs of clinical impact. Repeated positive cultures have been termed ‘persistent infection’ or ‘colonization,’ although the definition of persistent varies throughout the CF literature [15,17]. Hong et al. and Amin et al. defined persistence infection as isolation of *Aspergillus* at least twice in the preceding 12 months after initial identification. These studies have shown associations with *Aspergillus* and worse clinical outcome measures, yet these correlations require further investigation [20,21]. Prospective clinical studies involving fungal detection are needed to better understand the development and progression of lung disease and allergic phenotypes in people with CF and airway fungi.

Transient isolation of *Aspergillus* has been described, yet the clinical significance of this is not known and is often excluded from further analyses. Possible hypotheses for these findings of rare or infrequent fungus isolation may be microbiome shifts in the airway from antimicrobial use, environmental/occupational exposure, fungi being acquired with other common bacterial infections, or precursors to infection, ABPA, or allergic sensitization [15,17,23,65].

### 5.2. Fungal Bronchitis

In some patients, recovery of fungus appears to be associated with clinical disease [15,27,66]. Fungal bronchitis is often diagnosed and treated in the setting of incomplete or absent clinical response to weeks of antimicrobial therapy targeted at bacteria commonly responsible for pulmonary exacerbations. This in turn can lead to a delay in diagnosis or complicate the treatment regimen. Tracy and Moss provided an extensive review regarding the spectrum of fungal infection in pwCF, highlighting the complexity of active fungal infection with bronchitis, the possibility of persistent infection, and how colonization is present in this population with unknown significance [15]. Baxter et al. attempted to define fungal bronchitis further with elevated serological measurements of IgG to *Aspergillus* without IgE response to *Aspergillus*, as well as positive cultures, *A. fumigatus* specific PCR detection, and sputum galactomannan [17]. They found individuals with this serologic and clinical profile in 30% of the pwCF they studied, indicating a unique group of people responding to fungal infection [17].

Brandt et al. performed an extensive study using a case series, a prospective cohort study involving 22 subjects, and German registry data to further describe bronchitis caused by *Aspergillus* in pwCF [27]. In their prospective study, they used the definitions of fungal bronchitis defined by Baxter [17]. In their registry data, 32% of the population isolated *Aspergillus*; further, *Aspergillus* was associated with lower body-mass index (a measure of nutritional status), older age, and lower percent predicted forced expiratory volume at one second (ppFEV1). In their case review, they found those treated with itraconazole had significantly reduced cough and sputum production. Furthermore, using data from their smaller case series, they estimated a prevalence of *Aspergillus* bronchitis of 1.6% in the population; in their prospective cohort analysis of 22 subjects, the prevalence was higher with 9% (*n* = 2) developing *Aspergillus* bronchitis. While these studies attempt to identify markers of fungal infection, the clinical outcomes related to repeated recovery of *Aspergillus* remains to be elucidated.

Many studies have tried to identify a ‘chronic’ definition based upon frequency of positive airway cultures; however, criteria are inconsistent across studies and influenced by the number of cultures performed during the study period. Definitions have often been modeled after the Leeds criteria for chronic *Pseudomonas aeruginosa* infection, although a consensus has not been reached (e.g., 50% positive cultures in a year, two positive cultures in the past year) [15,16,17]. More prospective studies are needed to understand the impact of fungal infection frequency on clinical outcomes and in defining chronicity of infection.

### 5.3. Fungal Sensitization

There is some evidence that pwCF have higher rates of atopy and asthma, indicating a higher rate of sensitization to allergens in the environment. Studies have shown that adults with CF are more likely to be sensitized to multiple environmental fungal allergens, including ones that have not been cultured from their sputum, resulting in asthma like phenotypes [24,67,68]. This has not been extensively studied in pediatric patients and when increased rates of sensitization begin to develop is unclear.

### 5.4. Allergic Bronchopulmonary Aspergillosis

ABPA is an allergic disease related to fungal exposure in the airway [69]. Individuals with CF are at higher risk of ABPA, although the risk factors, exposures, and time frame are not completely understood. Individuals with CF and ABPA have been found to have lower lung function and more frequent co-morbidities, indicating a possible acceleration of the CF disease process [17,23].

The underlying pathophysiology of ABPA is thought to be an allergic response to antigens from the *Aspergillus sp.* organism. Diagnosis involves clinical worsening, radiographic changes on chest radiograph or computerized tomography (CT), elevation of total serum IgE to greater than 500 kUA/L, and positive IgG and IgE response to *Aspergillus* allergens. Treatment is complex and often prolonged, requiring long courses of oral steroids with tapering and sometimes antifungals as an adjuvant. Annual screening is recommended in pwCF starting in early childhood, as studies have shown a higher risk of ABPA in this population compared to others. Non-CF ABPA may be diagnosed in individuals with severe asthma and has slightly different diagnosis guidelines. Interestingly, a sputum culture that is positive for *Aspergillus* infection is not required for diagnosis, emphasizing that this is not necessarily active infection, but more so an allergic reaction to continued airway antigen exposure [17,23,24,69]. How a person with CF develops ABPA is unclear. A reaction to *Aspergillus* is needed, but whether this is genetic, environmental, or multifactorial is unclear. More studies are needed to elicit who is at risk for ABPA, what risk factors are associated with the diagnosis, and how to properly monitor and treat the condition.

## 6. Clinical Outcomes

People with CF and fungal infection have been shown to have lower lung function. Amin et al. performed a retrospective study looking at risk factors and lung function in patients with CF and *Aspergillus* infection [20]. In 230 patients, those persistently infected with *A. fumigatus* had 3.61% lower ppFEV1, with a significant association with *p. aeruginosa* infection when unadjusted for baseline ppFEV1. Furthermore, they found that those with persistent *A. fumigatus* infection had an increased risk for pulmonary exacerbation requiring hospitalization [20]. Sudfeld et al. performed a study on patients with CF and showed that with every 10% decrease in ppFEV1, the odds of isolating a filamentous fungus independently increased by 5% every quarter of a year [29]. However, there are studies that have shown no associations between *Aspergillus* and lung function [70]. While most work has been done in *Aspergillus*, decline in ppFEV1 has also been shown in pwCF isolating particular *Candida* species, a fungus not always reported or tested for in microbiology labs [71,72]. Despite these findings, evidence of causation is lacking.

Studies regarding *Aspergillus* and pulmonary exacerbations have also been mixed. Some studies showing transient *Aspergillus* isolation showed little difference in exacerbation frequency compared to those without *Aspergillus* [70]. Amin and al., described above, found increased risk for pulmonary exacerbation in those with persistent *Aspergillus* [20]. Efforts have also been made to study the impact of treatment of *Aspergillus* on pulmonary exacerbations. A double-blind randomized control trial comparing itraconazole treatment for *Aspergillus* infection in pwCF to placebo showed no difference in number of exacerbations or time to first exacerbation, yet the interpretation of these data was limited by post hoc determination of subtherapeutic drug levels and small sample size [73]. Conversely, one small study (*n* = 13) of pwCF and *Aspergillus* treated with 6 weeks of itraconazole showed reduced rates of respiratory symptoms and pulmonary exacerbations in those that cleared *Aspergillus* infection [74].

Studies have also shown some association with fungus and imaging findings in pwCF. The Australian Respiratory Early Surveillance Team for Cystic Fibrosis (AREST CF) group studied 310 children with 1035 CT scans and 1631 BAL samples for culture identification. They found increased rates of air trapping in pediatric patients with *Aspergillus* isolated from bronchoscopy as well as worse chest CT scores and progression of CT scores following fungal isolation [30]. The Australasian Cystic Fibrosis Bronchoalveolar Lavage study group (ACFBAL) confirmed this finding of air trapping in children at age 5 with CF and *Aspergillus* isolation, indicating possible airway edema, obstruction, or asthma like findings in pwCF and *Aspergillus* [31]. McMahon et al. reported similar findings, showing that pwCF and *Aspergillus* positive airway samples had more severe bronchiectasis on CT scan compared to those without *Aspergillus* [75].

Whether fungus in itself is causal or a bystander with another entity that causes these outcome differences has yet to be determined. More longitudinal and prospective studies are needed, as well as randomized controlled trials, to determine the effect of fungus on pwCF. As some studies have shown less isolation of *Aspergillus* following treatment with CFTR modulators, future approaches may differ. [63,76].

## 7. Co-Infection

Investigators have examined associations with fungus and other organisms found in the CF airway. Many of these studies have mixed results and lack clear cause and effect relationships between fungus and other infections. Nevertheless, several studies found that *P. aeruginosa* infection is associated with fungi detection [16,77]. Many theories exist regarding the timing of fungal infection related to *p*. *aeruginosa* and it is unclear if *Pseudomonas* infection precedes fungal infection or promotes fungal infection in some way. In multiple studies, those with both *Pseudomonas* and *Aspergillus* infection had worse clinical outcomes compared to other groups [20,78].

There is concern that antimicrobial use and microbiome shifts in the CF airway may allow fungus to thrive [18,49]. Studies have found associations between the treatment of *P*. *aeruginosa* and fungal detection following treatment, in particular following use of inhaled tobramycin [29,79]. This concept promotes the idea that reduction in *Pseudomonas* infection allows for less competition within CF sputum and airways, potentially providing biofilms and cell debris for fungus to utilize. 

There are some animal studies that have shown *Pseudomonas* biofilms inhibiting fungal growth, alluding to the possibility that active *Pseudomonas* infection may prevent fungal spores from germinating and causing infection [80,81]. Other studies found that biofilms created by *Pseudomonas* may in fact promote fungal infection. Animal and in vitro studies have shown *Aspergillus* thriving in the setting of *Pseudomonas* biofilms, theorizing that when germinating within these structures, fungal spores have the ability to further evade the host inflammatory response [58,82]. Other studies in adults have shown reduction in *Aspergillus* detection with the treatment of IV antibiotics, indicating a possible synergistic relationship amongst bacteria and fungi [83].

## 8. Inflammation in Fungal Infection

Infection in CF is generally thought to be a T-cell helper type one (Th1) process, characterized by a neutrophil driven T cell response, usually early in immune response. This is driven by interferon- gamma, macrophages, and neutrophil activation in the airway [5,84], and thought to be a reaction to simple, single-celled organisms, resulting in an inflammatory reaction in the airways. Studies have shown associations with Th1 markers and disease severity, showing changes in ppFEV1 and other outcome measures with markers of both systemic and organ specific inflammation [6,85]. Some studies have shown similar Th1 responses to fungus in pwCF. Studies analyzing BAL fluid have shown elevated IL8, neutrophil elastase (NE), and elevated neutrophil and total cell counts in those with *Aspergillus* infection [16,86]. Inflammation in CF is thought to result in permanent damage and remodeling to the airway, leading to airway obstruction and excessive mucous production. Over time, this can result in dependence on oxygen or positive pressure ventilation, pulmonary hypertension, and end stage lung disease requiring transplantation.

In contrast to the Th1 inflammatory response that generally characterizes CF lung disease, fungal infection in CF may generate a Th2 response. particularly in those with atopy or ABPA [24,60,67,87]. Th2 response is a T cell response that is thought to be later in activation and driven by a more allergic mechanism. It is often implicated in asthma, allergies, and parasite infections, showing an eosinophil predominant pathway of activation [60,88]. This has not been extensively studied in CF, yet given the predilection of ABPA there is concern that there may be a class switch, resulting in a more allergic response. Whether this occurs acutely, over time, or in genetically susceptible individuals still needs investigation.

**Figure 1:**
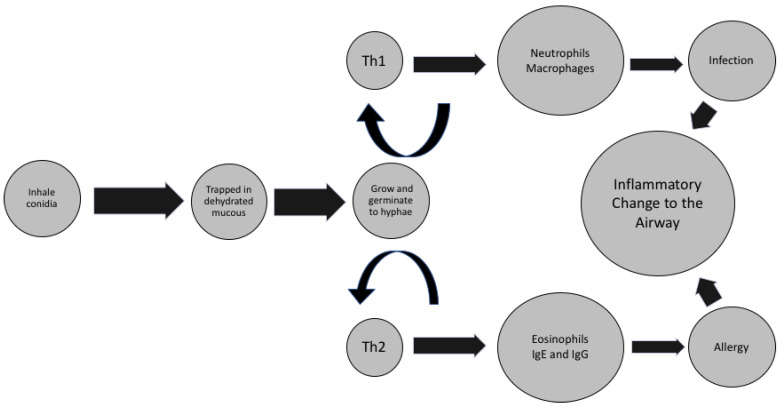
Possible inflammatory cycle in response to sustained exposure to fungus in CF airway, indicating role of Th1 and Th2 inflammation.

Th2 response is often thought of as being delayed compared to Th1, and for fungus to become infectious the organism needs to germinate and spawn over a prolonged period of time [60]. The CF mucus is likely to be an appropriate environment for this necessary time gap; it is thick, full of nutrients and cell products, warm, and isolated from inflammatory cells [2]. Given that there are concerns about a weak Th1 response to complex eukaryotic organisms, the delayed Th2 response could react to the complex fungal organisms/hyphae. If the Th2 response cannot kill it with the recruitment of eosinophils (basic pH secretions and chemicals), it could possibly promote ‘walling off’ of organisms within the airway. Since this response allows for a complex organism to grow, it thus could create more epitopes and peptides for antibodies and T cells to react to (Figure 1) [60,88].

While the host response and immune cells may eventually clear or suppress the infection, the complex antigens and peptides are still in the milieu of CF sputum and mucus in the airway. Thus, class switching to IgE and various other immunoglobulins may occur and promote an allergic-type response. This could result in eosinophil recruitment to fungal elements in the airway. Therefore, while pwCF may be able to clear the fungus itself, the re-exposure to elements may cause eosinophil and IgE responses, increased mucus production, and further activation of the inflammatory cascade.

Animal studies have shown possible Th2 responses to fungus in CF knockout animal models. Mice in these models showed impaired *Aspergillus* conidia uptake, ineffective clearance of *Aspergillus*, more inflammation, more neutrophils, eosinophils, and macrophages, and increased Th2 chemokines (MCP-1) [89]. Similar studies found elevated Th2 cytokines (IL13, IL5) with enhanced IgE response when focusing on lymphocyte defects in CF knock out mice, as well as little difference in IFN gamma, a Th1 cytokine [90,91].

More extensive human studies on the immune response to fungus are limited. One study focused on specific IL10 polymorphisms, a cytokine involved in Th2 processes, showing that certain genotypes have associations with *Aspergillus* as well as higher IL-10 levels [92]. Others have shown reduced Th2 responses to *Aspergillus* in patients with CF when supplemented with Vitamin D, which has been hypothesized as an immune modulator [93].

Further studies have shown atopic reactions to fungus in those with CF. Individuals with CF have been shown to have higher IgE levels and more robust reactions to fungal elements during skin testing when compared to healthy controls [67,68]. A meta-analysis by Maturu and Agarwal found a pooled prevalence of *Aspergillus* sensitization and ABPA of 39% and 8.9% respectively indicating a high prevalence of fungal sensitization in pwCF [94].

## 9. Future Directions

More work is needed to understand the role of fungus in CF (Table 1). As fungus is notably difficult to isolate from the airway, fungal infections may be underrepresented. Considerations for standardization of isolating fungus from sputum culture, BAL, qPCR methods, and throat swabs are necessary. The investigation of the microbiome and mycobiome also affords further insight to the extent of fungi in CF and its relationship to residing bacteria.

The effect of long-term fungal infection and risk factors for development of fungal infections needs further investigation. Fungus has been associated with disease progression and co-infection with *Pseudomonas*, but the pathobiology is unclear. More work is needed regarding whether fungus is causal in disease progression, synergistic with other pathogens in increasing morbidity, or a bystander indicative of some other developing disease or infectious process.

In the era of CFTR modulators, many aspects of CF care are changing. From our own experience, individuals with CF are producing less sputum while on highly-effective modulator therapy. Currently, sputum remains the prime medium for fungal detection, thus measuring the impact of CFTR modulation on fungal prevalence may be challenging. Novel systemic or non-invasively collected biomarkers of fungal disease will be critical for understanding fungal disease as CF management changes in the CFTR modulator era.

## Figures and Tables

**Table 1:** Future directions needed for the investigation of fungal disease in people with cystic fibrosis.

Area	Research Questions
Detection of fungi from airway samples	Improved culture approaches: media, optimal temperature, optimal timing, qPCR testing, mycobiome sequencing
Characterization of fungal infections	Chronicity of infection Classifications and outcomes (e.g., sporadic, intermittent, chronic)
Clinical Outcomes	Risk factors for worsening disease Management guidelines and indications for treatment Prospective, longitudinal studies needed
Co-Infection	Role of *Pseudomonas aeruginosa,* other gram-negative pathogens Biofilm development Antibiotic influence on fungi
Inflammation	Pathways and cell signaling Th1 versus Th2 host response Immunoglobulin shifts and class switching Identification of sputum and serum biomarkers
Allergic Bronchopulmonary Aspergillosis	Identification of risk factors Long term outcomes Genetic studies, role of atopic predisposition Sensitization and role of other fungi Response to novel therapies

## References

[1] Elborn J.S. (2016). Cystic fibrosis. Lancet.

[2] Rowe S.M., Miller S., Sorscher E.J. (2005). Cystic fibrosis. N. Engl. J. Med..

[3] Gibson R.L., Burns J.L., Ramsey B.W. (2003). Pathophysiology and management of pulmonary infections in cystic fibrosis. Am. J. Respir. Crit. Care Med..

[4] Burns J.L., Emerson J., Stapp J.R., Yim D.L., Krzewinski J., Louden L., Ramsey B.W., Clausen C.R. (1998). Microbiology of sputum from patients at cystic fibrosis centers in the United States. Clin. Infect. Dis..

[5] Cantin A.M., Hartl D., Konstan M.W., Chmiel J.F. (2015). Inflammation in cystic fibrosis lung disease: Pathogenesis and therapy. J. Cyst. Fibros..

[6] Sagel S.D., Wagner B.D., Anthony M.M., Emmett P., Zemanick E.T. (2012). Sputum biomarkers of inflammation and lung function decline in children with cystic fibrosis. Am. J. Respir. Crit. Care Med..

[7] Salsgiver E.L., Fink A.K., Knapp E.A., LiPuma J.J., Olivier K.N., Marshall B.C., Saiman L. (2016). Changing epidemiology of the respiratory bacteriology of patients with cystic fibrosis. Chest.

[8] Emerson J., Rosenfeld M., McNamara S., Ramsey B., Gibson R.L. (2002). *Pseudomonas aeruginosa* and other predictors of mortality and morbidity in young children with cystic fibrosis. Pediatr. Pulmonol..

[9] Mayer-Hamblett N., Kronmal R.A., Gibson R.L., Rosenfeld M., Retsch-Bogart G., Treggiari M.M., Burns J.L., Khan U., Ramsey B.W. (2012). Initial *Pseudomonas aeruginosa* treatment failure is associated with exacerbations in cystic fibrosis. Pediatr. Pulmonol..

[10] Sagel S.D., Gibson R.L., Emerson J., McNamara S., Burns J.L., Wagener J.S., Ramsey B.W. (2009). Impact of *Pseudomonas* and *Staphylococcus* infection on inflammation and clinical status in young children with cystic fibrosis. J. Pediatr..

[11] Wolter D.J., Emerson J.C., McNamara S., Buccat A.M., Qin X., Cochrane E., Houston L.S., Rogers G.B., Marsh P., Prehar K. (2013). *Staphylococcus aureus* small-colony variants are independently associated with worse lung disease in children with cystic fibrosis. Clin. Infect. Dis..

[12] Li Z., Kosorok M.R., Farrell P.M., Laxova A., West S.E., Green C.G., Collins J., Rock M.J., Splaingard M.L. (2005). Longitudinal development of mucoid Pseudomonas aeruginosa infection and lung disease progression in children with cystic fibrosis. JAMA.

[13] Sanders D.B., Bittner R.C., Rosenfeld M., Redding G.J., Goss C.H. (2011). Pulmonary exacerbations are associated with subsequent FEV1 decline in both adults and children with cystic fibrosis. Pediatr. Pulmonol..

[14] Goss C.H. (2019). Acute pulmonary exacerbations in cystic fibrosis. Semin. Respir. Crit. Care Med..

[15] Tracy M.C., Moss R.B. (2018). The myriad challenges of respiratory fungal infection in cystic fibrosis. Pediatric Pulmonol..

[16] King J., Brunel S.F., Warris A. (2016). *Aspergillus* infections in cystic fibrosis. J. Infect..

[17] Baxter C.G., Dunn G., Jones A.M., Webb K., Gore R., Richardson M.D., Denning D.W. (2013). Novel immunologic classification of aspergillosis in adult cystic fibrosis. J. Allergy Clin. Immunol..

[18] Botterel F., Angebault C., Cabaret O., Stressmann F.A., Costa J.M., Wallet F., Wallaert B., Bruce K., Delhaes L. (2018). Fungal and bacterial diversity of airway microbiota in adults with cystic fibrosis: Concordance between conventional methods and ultra-deep sequencing, and their practical use in the clinical laboratory. Mycopathologia.

[19] Coron N., Pihet M., Frealle E., Lemeille Y., Pinel C., Pelloux H., Gargala G., Favennec L., Accoceberry I., Durand-Joly I. (2018). Toward the standardization of mycological examination of sputum samples in cystic fibrosis: Results from a French multicenter prospective study. Mycopathologia.

[20] Amin R., Dupuis A., Aaron S.D., Ratjen F. (2010). The effect of chronic infection with *Aspergillus fumigatus* on lung function and hospitalization in patients with cystic fibrosis. Chest.

[21] Hong G., Alby K., Ng S.C.W., Fleck V., Kubrak C., Rubenstein R.C., Dorgan D.J., Kawut S.M., Hadjiliadis D. (2020). The presence of *Aspergillus fumigatus* is associated with worse respiratory quality of life in cystic fibrosis. J. Cyst. Fibros..

[22] Duesberg U., Wosniok J., Naehrlich L., Eschenhagen P., Schwarz C. (2020). Risk factors for respiratory *Aspergillus fumigatus* in German Cystic Fibrosis patients and impact on lung function. Sci. Rep..

[23] Janahi I.A., Rehman A., Al-Naimi A.R. (2017). Allergic bronchopulmonary aspergillosis in patients with cystic fibrosis. Ann. Thorac. Med..

[24] Antunes J., Fernandes A., Borrego L.M., Leiria-Pinto P., Cavaco J. (2010). Cystic fibrosis, atopy, asthma and ABPA. Allergol. Immunopathol..

[25] Delhaes L., Touati K., Faure-Cognet O., Cornet M., Botterel F., Dannaoui E., Morio F., Le Pape P., Grenouillet F., Favennec L. (2019). Prevalence, geographic risk factor, and development of a standardized protocol for fungal isolation in cystic fibrosis: Results from the international prospective study “MFIP”. J. Cyst. Fibros..

[26] Engel T.G.P., Slabbers L., de Jong C., Melchers W.J.G., Hagen F., Verweij P.E., Merkus P., Meis J.F. (2019). Prevalence and diversity of filamentous fungi in the airways of cystic fibrosis patients—A Dutch, multicentre study. J. Cyst. Fibros..

[27] Brandt C., Roehmel J., Rickerts V., Melichar V., Niemann N., Schwarz C. (2018). Aspergillus Bronchitis in Patients with Cystic Fibrosis. Mycopathologia.

[28] Hong G., Psoter K.J., Jennings M.T., Merlo C.A., Boyle M.P., Hadjiliadis D., Kawut S.M., Lechtzin N. (2018). Risk factors for persistent *Aspergillus* respiratory isolation in cystic fibrosis. J. Cyst. Fibros..

[29] Sudfeld C.R., Dasenbrook E.C., Merz W.G., Carroll K.C., Boyle M.P. (2010). Prevalence and risk factors for recovery of filamentous fungi in individuals with cystic fibrosis. J. Cyst. Fibros..

[30] Breuer O., Schultz A., Garratt L.W., Turkovic L., Rosenow T., Murray C.P., Karpievitch Y.V., Akesson L., Dalton S., Sly P.D. (2020). *Aspergillus* infections and progression of structural lung disease in children with cystic Fibrosis. Am. J. Respir. Crit. Care Med..

[31] Harun S.N., Wainwright C.E., Grimwood K., Hennig S. (2019). *Aspergillus* and progression of lung disease in children with cystic fibrosis. Thorax.

[32] Hong G., Lechtzin N., Hadjiliadis D., Kawut S.M. (2019). Inhaled antibiotic use is associated with *Scedosporium*/*Lomentospora* species isolation in cystic fibrosis. Pediatr. Pulmonol..

[33] Ziesing S., Suerbaum S., Sedlacek L. (2016). Fungal epidemiology and diversity in cystic fibrosis patients over a 5-year period in a national reference center. Med. Mycol..

[34] Blyth C.C., Middleton P.G., Harun A., Sorrell T.C., Meyer W., Chen S.C. (2010). Clinical associations and prevalence of *Scedosporium* spp. in Australian cystic fibrosis patients: Identification of novel risk factors?. Med. Mycol..

[35] Abdolrasouli A., Bercusson A.C., Rhodes J.L., Hagen F., Buil J.B., Tang A.Y.Y., de Boer L.L., Shah A., Milburn A.J., Elborn J.S. (2018). Airway persistence by the emerging multi-azole-resistant *Rasamsonia argillacea* complex in cystic fibrosis. Mycoses.

[36] Esther C.R., Plongla R., Kerr A., Lin F.C., Gilligan P. (2016). Clinical outcomes in cystic fibrosis patients with Trichosporon respiratory infection. J. Cyst. Fibros..

[37] Chen M., Kondori N., Deng S., Gerrits van den Ende A.H.G., Lackner M., Liao W., de Hoog G.S. (2018). Direct detection of *Exophiala* and *Scedosporium* species in sputa of patients with cystic fibrosis. Med. Mycol..

[38] Kondori N., Lindblad A., Welinder-Olsson C., Wenneras C., Gilljam M. (2014). Development of IgG antibodies to *Exophiala dermatitidis* is associated with inflammatory responses in patients with cystic fibrosis. J. Cyst. Fibros..

[39] Rosenfeld M., Emerson J., Accurso F., Armstrong D., Castile R., Grimwood K., Hiatt P., McCoy K., McNamara S., Ramsey B. (1999). Diagnostic accuracy of oropharyngeal cultures in infants and young children with cystic fibrosis. Pediatr. Pulmonol..

[40] Hoppe J.E., Towler E., Wagner B.D., Accurso F.J., Sagel S.D., Zemanick E.T. (2015). Sputum induction improves detection of pathogens in children with cystic fibrosis. Pediatr. Pulmonol..

[41] Ramsey B.W., Wentz K.R., Smith A.L., Richardson M., Williams-Warren J., Hedges D.L., Gibson R., Redding G.J., Lent K., Harris K. (1991). Predictive value of oropharyngeal cultures for identifying lower airway bacteria in cystic fibrosis patients. Am. Rev. Respir. Dis..

[42] Wainwright C.E., Vidmar S., Armstrong D.S., Byrnes C.A., Carlin J.B., Cheney J., Cooper P.J., Grimwood K., Moodie M., Robertson C.F. (2011). Effect of bronchoalveolar lavage-directed therapy on *Pseudomonas aeruginosa* infection and structural lung injury in children with cystic fibrosis: A randomized trial. JAMA.

[43] Saiman L., Siegel J., Cystic Fibrosis Foundation Consensus Conference on Infection Control Participants (2003). Infection control recommendations for patients with cystic fibrosis: Microbiology, important pathogens, and infection control practices to prevent patient-to-patient transmission. Infect. Control Hosp. Epidemiol..

[44] Chen S.C., Meyer W., Pashley C.H. (2018). Challenges in laboratory detection of fungal pathogens in the airways of cystic fibrosis patients. Mycopathologia.

[45] Ronchetti K., Tame J.D., Paisey C., Thia L.P., Doull I., Howe R., Mahenthiralingam E., Forton J.T. (2018). The CF-Sputum Induction Trial (CF-SpIT) to assess lower airway bacterial sampling in young children with cystic fibrosis: A prospective internally controlled interventional trial. Lancet Respir. Med..

[46] Burns J.L., Rolain J.M. (2014). Culture-based diagnostic microbiology in cystic fibrosis: Can we simplify the complexity?. J. Cyst. Fibros..

[47] Hong G., Miller H.B., Allgood S., Lee R., Lechtzin N., Zhang S.X. (2017). Use of selective fungal culture media increases rates of detection of fungi in the respiratory tract of cystic fibrosis patients. J. Clin. Microbiol..

[48] Delhaes L., Monchy S., Frealle E., Hubans C., Salleron J., Leroy S., Prevotat A., Wallet F., Wallaert B., Dei-Cas E. (2012). The airway microbiota in cystic fibrosis: A complex fungal and bacterial community--implications for therapeutic management. PLoS ONE.

[49] Nguyen L.D., Viscogliosi E., Delhaes L. (2015). The lung mycobiome: An emerging field of the human respiratory microbiome. Front. Microbiol..

[50] Cuthbertson L., Felton I., James P., Cox M.J., Bilton D., Schelenz S., Loebinger M.R., Cookson W.O.C., Simmonds N.J., Moffatt M.F. (2020). The fungal airway microbiome in cystic fibrosis and non-cystic fibrosis bronchiectasis. J. Cyst. Fibros..

[51] Toor A., Culibrk L., Singhera G.K., Moon K.M., Prudova A., Foster L.J., Moore M.M., Dorscheid D.R., Tebbutt S.J. (2018). Transcriptomic and proteomic host response to *Aspergillus fumigatus* conidia in an air-liquid interface model of human bronchial epithelium. PLoS ONE.

[52] Balloy V., Sallenave J.M., Wu Y., Touqui L., Latge J.P., Si-Tahar M., Chignard M. (2008). *Aspergillus fumigatus*-induced interleukin-8 synthesis by respiratory epithelial cells is controlled by the phosphatidylinositol 3-kinase, p38 MAPK, and ERK1/2 pathways and not by the toll-like receptor-MyD88 pathway. J. Biol. Chem..

[53] Zhang Z., Liu R., Noordhoek J.A., Kauffman H.F. (2005). Interaction of airway epithelial cells (A549) with spores and mycelium of *Aspergillus fumigatus*. J. Infect..

[54] Oosthuizen J.L., Gomez P., Ruan J., Hackett T.L., Moore M.M., Knight D.A., Tebbutt S.J. (2011). Dual organism transcriptomics of airway epithelial cells interacting with conidia of *Aspergillus fumigatus*. PLoS ONE.

[55] Gomez P., Hackett T.L., Moore M.M., Knight D.A., Tebbutt S.J. (2010). Functional genomics of human bronchial epithelial cells directly interacting with conidia of *Aspergillus fumigatus*. BMC Genom..

[56] Botterel F., Gross K., Ibrahim-Granet O., Khoufache K., Escabasse V., Coste A., Cordonnier C., Escudier E., Bretagne S. (2008). Phagocytosis of *Aspergillus fumigatus* conidia by primary nasal epithelial cells in vitro. BMC Microbiol..

[57] Williams C., Ranjendran R., Ramage G. (2016). Pathogenesis of fungal infections in cystic fibrosis. Curr. Fungal Infect. Rep..

[58] Kaur S., Singh S. (2014). Biofilm formation by *Aspergillus fumigatus*. Med. Mycol..

[59] Yoon S.S., Hennigan R.F., Hilliard G.M., Ochsner U.A., Parvatiyar K., Kamani M.C., Allen H.L., DeKievit T.R., Gardner P.R., Schwab U. (2002). Pseudomonas aeruginosa anaerobic respiration in biofilms: Relationships to cystic fibrosis pathogenesis. Dev. Cell.

[60] Stevens D.A. (2006). Th1/Th2 in aspergillosis. Med. Mycol..

[61] Lopes-Pacheco M. (2019). CFTR modulators: The changing face of cystic fibrosis in the era of precision medicine. Front. Pharmacol..

[62] Donaldson S.H., Laube B.L., Corcoran T.E., Bhambhvani P., Zeman K., Ceppe A., Zeitlin P.L., Mogayzel P.J., Boyle M., Locke L.W. (2018). Effect of ivacaftor on mucociliary clearance and clinical outcomes in cystic fibrosis patients with G551D-CFTR. JCI Insight.

[63] Heltshe S.L., Mayer-Hamblett N., Burns J.L., Khan U., Baines A., Ramsey B.W., Rowe S.M. (2015). *Pseudomonas aeruginosa* in cystic fibrosis patients with G551D-CFTR treated with ivacaftor. Clin. Infect. Dis..

[64] Bercusson A., Jarvis G., Shah A. (2021). CF fungal disease in the age of CFTR modulators. Mycopathologia.

[65] Sabino R., Verissimo C., Viegas C., Viegas S., Brandao J., Alves-Correia M., Borrego L.M., Clemons K.V., Stevens D.A., Richardson M. (2019). The role of occupational *Aspergillus* exposure in the development of diseases. Med. Mycol..

[66] Shoseyov D., Brownlee K.G., Conway S.P., Kerem E. (2006). *Aspergillus* bronchitis in cystic fibrosis. Chest.

[67] Henry M., Bennett D.M., Kiely J., Kelleher N., Bredin C.P. (2000). Fungal atopy in adult cystic fibrosis. Respir. Med..

[68] Tobin M.J., Maguire O., Reen D., Tempany E., FitzGerald M.X. (1980). Atopy and bronchial reactivity in older patients with cystic fibrosis. Thorax.

[69] Moss R.B. (2010). Allergic bronchopulmonary aspergillosis and *Aspergillus* infection in cystic fibrosis. Curr. Opin. Pulm. Med..

[70] De Vrankrijker A.M., van der Ent C.K., van Berkhout F.T., Stellato R.K., Willems R.J., Bonten M.J., Wolfs T.F. (2011). *Aspergillus fumigatus* colonization in cystic fibrosis: Implications for lung function?. Clin. Microbiol. Infect..

[71] AbdulWahab A., Salah H., Chandra P., Taj-Aldeen S.J. (2017). Persistence of *Candida dubliniensis* and lung function in patients with cystic fibrosis. BMC Res. Notes.

[72] Al Shakirchi M., Klingspor L., Bergman P., Hjelte L., de Monestrol I. (2020). A 16-year retrospective study on fungal prevalence and diversity in patients with cystic fibrosis: *Candida dubliniensis* was associated with a decline in lung function. Int. J. Infect. Dis..

[73] Aaron S.D., Vandemheen K.L., Freitag A., Pedder L., Cameron W., Lavoie A., Paterson N., Wilcox P., Rabin H., Tullis E. (2012). Treatment of *Aspergillus fumigatus* in patients with cystic fibrosis: A randomized, placebo-controlled pilot study. PLoS ONE.

[74] Coughlan C.A., Chotirmall S.H., Renwick J., Hassan T., Low T.B., Bergsson G., Eshwika A., Bennett K., Dunne K., Greene C.M. (2012). The effect of *Aspergillus fumigatus* infection on vitamin D receptor expression in cystic fibrosis. Am. J. Respir. Crit. Care Med..

[75] McMahon M.A., Chotirmall S.H., McCullagh B., Branagan P., McElvaney N.G., Logan P.M. (2012). Radiological abnormalities associated with *Aspergillus* colonization in a cystic fibrosis population. Eur. J. Radiol..

[76] Frost F.J., Nazareth D.S., Charman S.C., Winstanley C., Walshaw M.J. (2019). Ivacaftor is associated with reduced lung infection by key cystic fibrosis pathogens. A cohort study using national registry data. Ann. Am. Thorac. Soc..

[77] Keown K., Reid A., Moore J.E., Taggart C.C., Downey D.G. (2020). Coinfection with Pseudomonas aeruginosa and Aspergillus fumigatus in cystic fibrosis. Eur. Respir. Rev..

[78] Reece E., Segurado R., Jackson A., McClean S., Renwick J., Greally P. (2017). Co-colonisation with *Aspergillus fumigatus* and *Pseudomonas aeruginosa* is associated with poorer health in cystic fibrosis patients: An Irish registry analysis. BMC Pulm. Med..

[79] Burns J.L., Van Dalfsen J.M., Shawar R.M., Otto K.L., Garber R.L., Quan J.M., Montgomery A.B., Albers G.M., Ramsey B.W., Smith A.L. (1999). Effect of chronic intermittent administration of inhaled tobramycin on respiratory microbial flora in patients with cystic fibrosis. J. Infect. Dis..

[80] Mowat E., Rajendran R., Williams C., McCulloch E., Jones B., Lang S., Ramage G. (2010). *Pseudomonas aeruginosa* and their small diffusible extracellular molecules inhibit *Aspergillus fumigatus* biofilm formation. FEMS Microbiol. Lett..

[81] Kaur J., Pethani B.P., Kumar S., Kim M., Sunna A., Kautto L., Penesyan A., Paulsen I.T., Nevalainen H. (2015). *Pseudomonas aeruginosa* inhibits the growth of *Scedosporium aurantiacum*, an opportunistic fungal pathogen isolated from the lungs of cystic fibrosis patients. Front. Microbiol..

[82] Zhao J., Yu W. (2018). Interaction between *Pseudomonas aeruginosa* and *Aspergillus fumigatus* in cystic fibrosis. PeerJ.

[83] Baxter C.G., Rautemaa R., Jones A.M., Webb A.K., Bull M., Mahenthiralingam E., Denning D.W. (2013). Intravenous antibiotics reduce the presence of *Aspergillus* in adult cystic fibrosis sputum. Thorax.

[84] Sagel S.D., Chmiel J.F., Konstan M.W. (2007). Sputum biomarkers of inflammation in cystic fibrosis lung disease. Proc. Am. Thorac. Soc..

[85] Sagel S.D., Kapsner R.K., Osberg I. (2005). Induced sputum matrix metalloproteinase-9 correlates with lung function and airway inflammation in children with cystic fibrosis. Pediatr. Pulmonol..

[86] Gangell C., Gard S., Douglas T., Park J., de Klerk N., Keil T., Brennan S., Ranganathan S., Robins-Browne R., Sly P.D. (2011). Inflammatory responses to individual microorganisms in the lungs of children with cystic fibrosis. Clin. Infect. Dis..

[87] Kent B.D., Lane S.J., van Beek E.J., Dodd J.D., Costello R.W., Tiddens H.A. (2014). Asthma and cystic fibrosis: A tangled web. Pediatr. Pulmonol..

[88] Schuyler M. (1998). The Th1/Th2 paradigm in allergic bronchopulmonary aspergillosis. J. Lab. Clin. Med..

[89] Chaudhary N., Datta K., Askin F.B., Staab J.F., Marr K.A. (2012). Cystic fibrosis transmembrane conductance regulator regulates epithelial cell response to *Aspergillus* and resultant pulmonary inflammation. Am. J. Respir. Crit. Care Med..

[90] Allard J.B., Poynter M.E., Marr K.A., Cohn L., Rincon M., Whittaker L.A. (2006). *Aspergillus fumigatus* generates an enhanced Th2-biased immune response in mice with defective cystic fibrosis transmembrane conductance regulator. J. Immunol..

[91] Muller C., Braag S.A., Herlihy J.D., Wasserfall C.H., Chesrown S.E., Nick H.S., Atkinson M.A., Flotte T.R. (2006). Enhanced IgE allergic response to *Aspergillus fumigatus* in CFTR-/- mice. Lab. Investig..

[92] Brouard J., Knauer N., Boelle P.Y., Corvol H., Henrion-Caude A., Flamant C., Bremont F., Delaisi B., Duhamel J.F., Marguet C. (2005). Influence of interleukin-10 on *Aspergillus fumigatus* infection in patients with cystic fibrosis. J. Infect. Dis..

[93] Nguyen N.L., Pilewski J.M., Celedon J.C., Mandalapu S., Blanchard M.L., DeRicco A., Hartigan E., Alcorn J.F., Kolls J.K. (2015). Vitamin D supplementation decreases *Aspergillus fumigatus* specific Th2 responses in CF patients with aspergillus sensitization: A phase one open-label study. Asthma Res. Pract..

[94] Maturu V.N., Agarwal R. (2015). Prevalence of Aspergillus sensitization and allergic bronchopulmonary aspergillosis in cystic fibrosis: Systematic review and meta-analysis. Clin. Exp. Allergy.

